# Genomic conflicts and sexual antagonism in human health: insights from oxytocin and testosterone

**DOI:** 10.1111/eva.12244

**Published:** 2015-02-04

**Authors:** Mikael Mokkonen, Bernard J Crespi

**Affiliations:** 1 Department of Biological Sciences, Simon Fraser UniversityBurnaby, BC, Canada; 2 Department of Biological and Environmental Science, University of JyväskyläJyväskylä, Finland

**Keywords:** genomic imprinting, kinship theory, parental antagonism, parent–offspring conflict, sexual antagonism, sexual conflict

## Abstract

We review the hypothesized and observed effects of two of the major forms of genomic conflicts, genomic imprinting and sexual antagonism, on human health. We focus on phenotypes mediated by peptide and steroid hormones (especially oxytocin and testosterone) because such hormones centrally mediate patterns of physical and behavioral resource allocation that underlie both forms of conflict. In early development, a suite of imprinted genes modulates the human oxytocinergic system as predicted from theory, with paternally inherited gene expression associated with higher oxytocin production, and increased solicitation to mothers by infants. This system is predicted to impact health through the incompatibility of paternal-gene and maternal-gene optima and increased vulnerability of imprinted gene systems to genetic and epigenetic changes. Early alterations to oxytocinergic systems have long-term negative impacts on human psychological health, especially through their effects on attachment and social behavior. In contrast to genomic imprinting, which generates maladaptation along an axis of mother–infant attachment, sexual antagonism is predicted from theory to generate maladaptation along an axis of sexual dimorphism, modulated by steroid and peptide hormones. We describe evidence of sexual antagonism from studies of humans and other animals, demonstrating that sexually antagonistic effects on sex-dimorphic phenotypes, including aspects of immunity, life history, psychology, and behavior, are commonly observed and lead to forms of maladaptation that are demonstrated, or expected, to impact human health. Recent epidemiological and psychiatric studies of schizophrenia in particular indicate that it is mediated, in part, by sexually antagonistic alleles. The primary implication of this review is that data collection focused on (i) effects of imprinted genes that modulate the oxytocin system, and (ii) effects of sexually antagonistic alleles on sex-dimorphic, disease-related phenotypes will lead to novel insights into both human health and the evolutionary dynamics of genomic conflicts.

## Introduction

Genomic conflicts are expected to be frequent sources of phenotypic maladaptation, as the optima targeted by different genomic agents may be more or less displaced from the values that maximize survival and reproduction at the level of organisms themselves (Burt and Trivers 2006). For humans, such displacements from fitness maximizing trajectories are expected to frequently manifest as deviations from health, given that alleles associated with greater disease susceptibility are costly for fitness through reduced survival or impaired reproduction. Any such deviations, moreover, should be structured by the nature and directions of genomic conflicts (Frank and Crespi 2011) such that better understanding of these conflicts, and their ongoing natures or potential resolutions, provides important insights into how to understand, prevent, and treat conflict-relevant diseases.

In this article, we provide a review and synthesis of two major forms of genomic conflicts, genomic imprinting, and sexual antagonism, with regard to their impacts on hormonally mediated, health-related human phenotypes. These two forms of genomic conflict are fundamentally similar in their expected impacts on health and disease, because they both centrally involve different fitness optima for two parties, and they both engender conflicts over optima that can involve the evolution of conflict mechanisms and evolutionary escalation; in both cases, disease-related impacts may ensue due to the deviations from optima and dysregulation of evolved conflict systems (Crespi 2010a; Frank and Crespi 2011). These forms of conflict also encompass two of the major forms of social interactions among sexual organisms, parents with offspring and males with females. We focus mainly on the hormones oxytocin and testosterone due to intense research interest in their physical and psychological effects, their wide-ranging influences on core aspects of development, cognition, and reproduction, and the central roles of oxytocin in mother–offspring interactions and testosterone in male–female differences.

We first discuss how hormones in general, and oxytocin and testosterone in particular, function in the context of evolved systems that mediate phenotypic adaptation and trade-offs. Second, we explain genomic imprinting and sexual antagonism, with regard to salient evolutionary theory, methods for detection and quantification of their effects, examples from the literature from animals and humans, and documented or postulated impacts on human health. In these contexts, we also discuss the similarities and differences between these two forms of conflict. We conclude with suggestions for future studies that integrate the evolutionary theory of genomic conflicts with health applications, especially for hormonally mediated traits. Although we focus predominantly on humans, the considerations below also apply to other sexual organisms, and taxa with genomic imprinting (mainly mammals and flowering plants), and to fitness-related impacts of genomic conflicts other than health and disease.

## Functions of oxytocin and testosterone

Oxytocin, a peptide hormone, and testosterone, a steroid hormone, represent two of the best studied endocrine mediators of development, cognition, and behavior in humans and other mammals (McCall and Singer 2012; Auyeung et al. 2013). As such, these hormones exhibit relatively well-understood, as well as pervasive, effects on human adaptive functioning, such that links to maladaptation and disease can be more readily hypothesized and forged.

Oxytocin is produced mainly in the hypothalamus and is released into both the brain, where it modulates neurotransmission, and the peripheral circulation, where it regulates parturition, lactation, and other physiological processes (Gimpl and Fahrenholz 2001; Knobloch and Grinevich 2014). In both settings, oxytocin exerts its effects via binding to oxytocin receptors and stimulation of intracellular signaling. Effects of oxytocin in the brain include modulation of maternal behavior, social bonding, sexual and agonistic behavior, feeding, stress, and anxiety, which are organized via the distributions and densities of oxytocin receptors (Donaldson and Young 2008; Lee et al. 2009; Anacker and Beery 2013; Carter 2014). Neurologically, oxytocin can be considered as a hormonal mediator of social behavior and cognition, through its effects on social attention, social salience (perceived importance of social events), social motivation, social bonding, and social reward (Bethlehem et al. 2014). Early-life behavioral events, especially in infancy, appear to exert ‘organizational’ effects on the oxytocinergic system, mediating sensitivity to its effects (Feldman et al. 2013), although such processes have yet to be clearly elucidated in humans or other mammals. Most generally, increases and decreases in oxytocin, and receptor densities, within different regions of the brain modulate condition-dependent, sex-dependent, and context-dependent behavioral propensities and choices related to social interactions (Hammock and Young 2006). Higher oxytocin activity is thus associated with increased focus on social stimuli and, usually, increased prosocial behavior in the contexts of interactions within one's own social groups of kin and non-kin, which include parents and offspring, male–female pair bonds, and larger groups defined by kinship or other societal factors (De Dreu 2012; Goodson 2013; Carter 2014). Oxytocin is expected to be of particular importance in humans compared to other animals, given the high levels of complexity to human social interaction and behavior.

Testosterone is produced in the testis, adrenal cortex, and (in females) ovaries, with bodily functions, of course, in the development of male primary and secondary sexual characteristics through activation of androgen receptors. With regard to brain and behavior, prenatal and early postnatal testosterone exerts organizational effects on neurodevelopment and cognitive functions (Auyeung et al. 2013). By comparison, after sexual maturation, such effects center on modulation by testosterone of energetic and time investment trade-offs between mating effort (higher levels) and parental effort (lower levels) in males, as well as trade-offs with investment in other domains such as immune functions (e.g., Klein 2000; Muehlenbein and Bribiescas 2005). In this context, sexual motivation, intrasexual dominance, success in male-typical endeavors, and social status represent primary determinants and arbitrators of success in mating effort, especially for males.

In contrast to oxytocin, testosterone is associated relatively strongly with ‘proself’ and self-oriented, rather than prosocial and other-oriented, cognition and behavior (Wright et al. 2012). This difference between oxytocin and testosterone is reflected in opposite effects of these two hormones for a remarkable range of phenotypes, including trust (Bos et al. 2010, 2012; Van IJzendoorn and Bakermans-Kranenburg 2012), empathy (Domes et al. 2007a,b; van Honk et al. 2011), parenting (Gettler et al. 2011; Feldman et al. 2012; Okabe et al. 2013; Weisman et al. 2014), and amygdala connectivity with social-brain regions such as the orbitofrontal cortex (van Wingen et al. 2010; Volman et al. 2011; Bos et al. 2013; Sripada et al. 2013). Most broadly, these diametric effects frame our consideration of the primary adaptive functions, and maladaptive effects in disease, of these two hormones, as reflecting an axis of social interaction (for oxytocin), compared to an axis of self-orientation, sexuality, and gender (for testosterone). This is a considerable oversimplification, given the interacting roles of other hormones such as estrogens, arginine vasopressin, and cortisol (e.g., van Anders et al. 2011), but it serves as a useful and pragmatic first step in developing the connections of endocrine adaptations with the genetics, and epigenetics, of human risks and manifestations of disease. This conceptualization also structures the contexts and importance of these hormones regarding genomic conflicts, which should thus also be aligned with social, and sexual, conflicts and confluences of interest.

## Genomic imprinting

**Figure 1:**
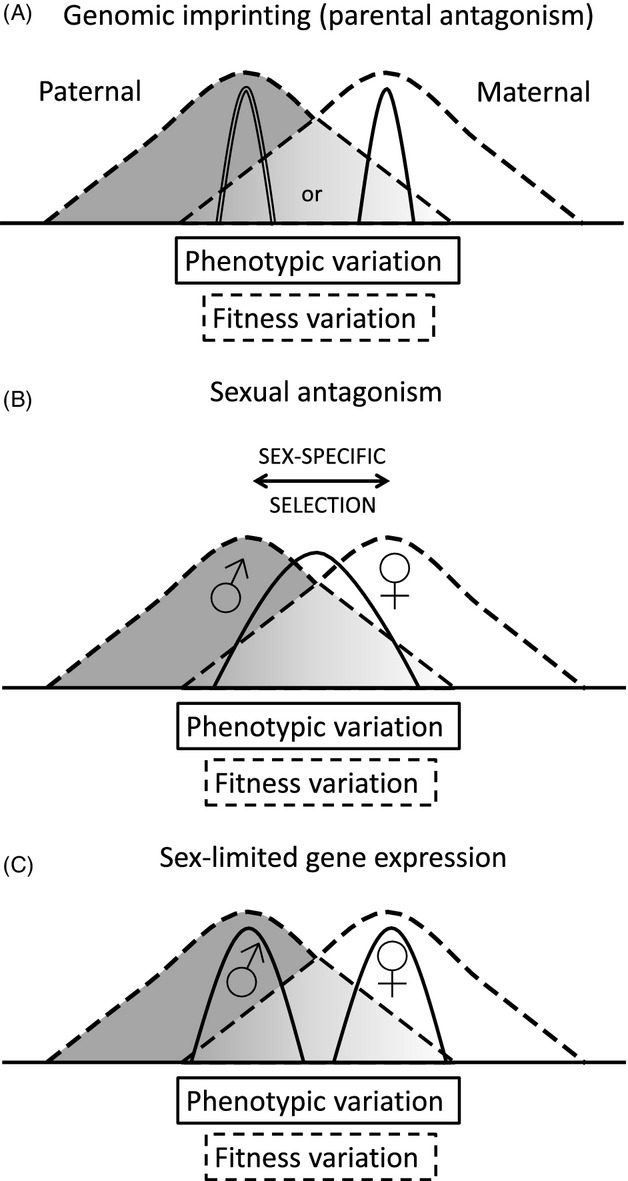
(A) A model of phenotypes mediated by genomic imprinting (parental antagonism). Under imprinting, one parent silences an allele during gametogenesis, thereby gaining a short-term fitness advantage. Subsequent selection on expression of the allele from the other parent leads to optimization of gene expression for that parent, for this locus. Resolution via joint optimization is not feasible in this system, so deleterious effects on health of one or both parties are expected unless multiple paternal and maternal loci exert effects, on the same phenotype, that balance. (B) A model of phenotypes experiencing sexual antagonism. The dashed lines indicate the fitness surfaces for females and males, respectively, and the single solid line indicates the distribution of phenotypic variation for the population. Deviations from sex-specific optima are expected to engender proportional deleterious effects on sex-specific health. (C) Distributions of phenotypic traits resulting from sex-limited gene expression, with sexual antagonism resolved. The dashed lines indicate the fitness surface for females and males, respectively, the single solid line indicates phenotypic variation for the population, with clear sexual dimorphism, and health effects from conflict are not predicted except from new sexually antagonistic mutations.

Genomic imprinting refers to silencing of an allele in an offspring according to its parent of origin, either the mother or father (Fig.1A). Genomic imprinting has evolved under conditions of higher relatedness to maternal kin compared to paternal kin, coupled with increased investment in offspring by females compared to males (Haig 2004, 2013). Usually, such higher relatedness among maternal kin than paternal kin is due to an evolutionary history of higher multiple paternity than multiple maternity within and across broods. By the well-supported kinship theory of imprinting (Ubeda and Haig 2003; Wilkins and Haig 2003; Haig 2013), and for mother–offspring interactions, lower relatedness through paternally inherited than maternally inherited alleles has selected for silencing in fathers (in developing sperm cell lineages) of genes that reduce maternal investment, which leads to higher levels of ‘selfish’ solicitation of resources from the mother, by offspring. In turn, selection in mothers has favored silencing (in developing oocytes) of other genes, specifically those whose expression in offspring leads to increased maternal investment through higher degrees of offspring solicitation of resources or other means. Imprinting effects are predicted in any situations involving relatedness asymmetries between maternal and paternal kin, but such parent–offspring interactions appear to represent their primary selective context throughout mammalian evolution.

Imprinted genes are expected to be relatively strongly associated with maladaptation and risk of disease, compared to other genes, for several reasons. First, their haploid expression pattern exposes novel, deleterious alleles to selection; moreover, under haploidy, dysregulation can relatively easily lead to (approximate) doubling or loss of expression, as commonly observed during carcinogenesis and some disorders of fetal and childhood growth (Horsthemke 2014). Second, imprinting leads to the evolution of new, conflict-related molecular mechanisms (such as ligand traps and antisense RNA transcripts) that represent novel targets for genetic or epigenetic dysregulation. Third, alterations to imprinted genes are expected to generate especially strong and direct impacts on fitness (and thus health), given that they have evolved squarely in the context of strongly fitness-related interactions of, for example, mothers with offspring. Fourth, imprinting-related conflicts can generate situations where both parties are wasting important fitness-related resources, but deviations from the equilibrium are jointly disfavored. Finally, the conflictual nature of imprinted gene systems necessarily produces trade-offs in fitness between offspring and mothers; if one party ‘wins’, the other is maximally displaced from its optimum (Fig.1A), and any intermediate, more or less stable resolution involves the same total displacements from fitness optima distributed across both parties.

Well-established associations of imprinted genes with disorders of placental development, prenatal and postnatal growth, risk of type 2 diabetes, cancer, and psychiatric conditions (e.g., Kong et al. 2009; Peters 2014) have been reported, although human disease-genetic studies of common or rare variants have yet to focus on imprinted genes in any systematic or comprehensive manner. Given the central roles of oxytocin in mother–offspring interactions described above, and human social behavior much more generally, do genomic imprinting effects impact this hormonal system, and if so how?

## Oxytocin, genomic imprinting, and human disease

Effects of imprinted genes on mammalian phenotypes and disease have been measured predominantly by analyzing the effects of gene knockouts or knockins in mice, characterizing phenotypes associated with single nucleotide polymorphisms in imprinted genes, and by studying the effects in humans of imprinting disorders due to deletions or duplications of imprinted regions, or epimutations, that generate so-called imprinted gene syndromes (Peters 2014).

A central, key aspect of early postnatal brain and cognitive development is attachment to the mother, which mediates both feeding and social-psychological development largely through the hormonal effects of oxytocin in both the developing child and the mother. As the discovery that behavioral and mating system variation in some rodents is associated with expression and reactivity to oxytocin and the closely related neuropeptide arginine vasopressin, the roles of oxytocin in human psychology, social behavior, and disease have been of increasing interest (Bethlehem et al. 2014; Carter 2014). Despite such concentrated attention on oxytocin's effects, no previous studies have considered the evidence, and implications for health, regarding genomic imprinting and genomic conflicts in the oxytocinergic systems of rodents or humans, despite the well-established importance of imprinted genes in mother–offspring interactions (Isles and Holland 2005; Crespi 2010b, 2011).

Genomic imprinting effects center strongly on conflict over solicitation or demands imposed by offspring on the mother. Given that oxytocin produced in offspring drives bonding and attachment to the mother (Feldman et al. 2013; Olff et al. 2013), an essential prerequisite to both energetic and psychological maternal-resource acquisition, a strong prediction from theory is that paternally expressed (maternally silenced) imprinted genes should favor increased offspring oxytocin production and increased sensitivity via effects on receptor systems. Moreover, higher levels of paternally expressed imprinted gene products, and higher oxytocin levels in offspring, should be associated with increased levels of offspring sucking, and faster postnatal growth. Conversely, maternal biases to imprinted gene expression, as from reductions in paternally expressed imprinted gene products, should lead to reduced bonding and attachment, less sucking, and slower growth. Such effects during infancy are predicted to have especially pervasive effects on health given fetal programming of adult disease risks and the highly pleiotropic effects of oxytocin in physiological and psychological development.

Table1 summarizes the available evidence from comprehensive searches of the relevant literature focusing on imprinted genes relating to oxytocin, offspring sucking, and postnatal growth. Four imprinted genes, PEG3, NDN, MAGEL2, and DLK1, which are all paternally expressed, are associated with oxytocin, feeding, and growth as predicted from theory, in mice, humans, or both. In all four cases, knockouts are known (PEG3, NDN, MAGEL2), or expected (DLK1), to exhibit decreases in levels of oxytocin-secreting neurons in the hypothalamus, apparently through more or less selective reductions in these cell populations. These parallel findings fit closely with theory and are especially interesting in the context of the central, highly connected hub positions of PEG3 and NDN genes in the imprinted gene coexpression network inferred by Varrault et al. (2006) in mice. Based on the documented behavioral effects of oxytocin, large-scale reductions in hypothalamic oxytocin-secreting neurons are expected to lead to reduced solicitation of resources by offspring, both energetic (sucking) and behavioral (huddling for warmth and physical contact such as licking and grooming). Reduced solicitation of maternal contact (via activity and crying) and reduced feeding motivation in infancy have also been reported as primary features of the human equivalents of paternal ‘knockouts’ of the NDN and MAGEL2 genes (as well as other genes), Prader–Willi syndrome, which also involves reductions in oxytocinergic neurons in the hypothalamus (Swaab et al. 1995). Reduced expression of DLK1 (most commonly due to maternal uniparental disomy: bearing two maternal copies of chromosome 14) also leads to a set of features, including infant postnatal growth reductions, that overlaps with those of Prader–Willi syndrome (Cox et al. 2004; Crespi 2008), with comparable phenotypes also found in DLK1-knockout mice (Moon et al. 2002).

**Table 1:** Evidence for effects of genomic inprinting on the oxytocinergic system and infant sucking. This table presents all of the data on imprinted genes in this context, and there are no findings that contravene expectations from theory

Imprinted gene, expression pattern	Evidence regarding oxytocin, infant feeding	References
PEG3 (paternally- expressed gene 3)	Knockout associated with reduced sucking in pups, large reduction in oxytocinergic neurons in adult females, reduced nursing by mothers, in mice	Li et al. (1999), Curley et al. (2004) and Champagne et al. (2009)
NDN (necdin), paternally-expressed	Knockout associated with large reduction in oxytocinergic neurons in hypothalamus, in mice	Muscatelli et al. (2000)
MAGEL2 (MAGE-like 2), paternally-expressed	Knockouts show poor infant sucking, large reduction in oxytocinergic neurons in hypothalamus, impaired oxytocin secretion; knockouts rescued by single postnatal oxytocin injection, in mice	Schaller et al. (2010) ans Schaaf et al. (2013)
GNASxl locus (Guanine Nucleotide-Binding Protein G(S) Subunit Alpha Isoforms XLas, paternally-expressed	Knockouts show reduced sucking in mice, deletions involve reduced sucking in humans	Plagge et al. (2004), Geneviève et al. (2005)
DLK1 (Delta-like 1 Homolog), paternally-expressed	Highly, selectively expressed in oxytocinergic neurons of hypothalamus after birth, in mice, and affects post-natal growth; locus appears to underlie feeding reductions in human maternal uniparental disomy of chromosome 14	Buiting et al. (2008), Temple et al. (2009) and Villanueva et al. (2012)
GTF2I (General Transcription Factor IIi), maternal bias in expression	Williams syndrome involves deletion of one copy, increased levels of serum oxytocin, increased social behavior; duplications involve separation anxiety in both mice and humans; unknown if gene affects oxytocinergic system	Collette et al. (2009), Dai et al. (2012) and Mervis et al. (2012)
PEG1 (MEST) Paternally-expressed gene 1	Knockout females show reduced, abnormal maternal behavior, comparable to that of PEG3 knockouts; effects on feeding, oxytocinergic system, unstudied	Lefebvre et al. (1998)

This convergent set of findings, across genes (PEG3, NDN, MAGEL2, DLK1) and species (humans and mice), is important for human health in at least three ways. First, these data indicate the existence of a set of imprinted genes that impacts directly and strongly on infant feeding and mother–infant bonding-related behavior. The degree to which segregating genetic and epigenetic variation in such genes modulates these phenotypes in humans, however, remains unstudied.

Second, the oxytocinergic system apparently originated in mammals in the context of viviparity, lactation, and mother–offspring bonding, but has since come to be co-opted for bonding between mates and between members of a cooperating social group (Gimpl and Fahrenholz 2001; Anacker and Beery 2013). As a result, alterations to imprinted genes that affect the oxytocin system are expected to have highly pleiotropic effects on human social behavior and psychology, throughout the life span. For example, in mice, knockouts of PEG3 and PEG1/MEST both lead to decreases in maternal behavior (among adult knockout females), including impaired lactation and pup retrieval (Lefebvre et al. 1998; Li et al. 1999), which can most parsimoniously be explained as secondary, long-term, purely maladaptive effects of reduced hypothalamic oxytocin secretion, given that this hormone orchestrates maternal care and feeding of offspring as well as offspring bonding to the mother. In humans, allelic variation in nonimprinted oxytocinergic genes including OXTR modulates the quality of maternal care of infants (e.g., Feldman et al. 2013), but imprinted genes have yet to be studied in this behavioral context. However, Prader–Willi syndrome and related conditions that affect hypothalamic development involve insecure psychological childhood attachment to mothers and represent strong risk factors for psychosis in adulthood (Crespi 2008; Soni et al. 2008). Oxytocin is also considered to be an important hormonal mediator of risk for other psychiatric conditions such as autism, given its general roles in promoting social attention and bonding (Cochran et al. 2013).

Third, the oxytocin system regulates feeding and satiety, especially for foods with high hedonic value (such as chocolate chip cookies, in one study; Ott et al. 2013). In mammals, feeding is naturally a social behavior motivated by reward (with strong links to the dopaminergic system and nucleus accumbens ‘pleasure center’) (Sabatier et al. 2013). Indeed, in dairy cows, levels of plasma oxytocin in calves are substantially higher if they suckle from their mother than if they drink the same milk from a bucket (Lupoli et al. 2001). These findings, strong links between disturbances to the oxytocin system and obesity (Chaves et al. 2013; Sabatier et al. 2013) (as found, for example, in Prader–Willi syndrome and maternal uniparental disomy 14), and the role of the oxytocin-dopamine system in addiction (Tops et al. 2014), indicate that imprinted genes mediating oxytocin release and responsivity are also expected to show pleiotropic, conflict-associated effects in these important health-related contexts.

The situations described above all involve experimental, mutational, or epimutational reduction of oxytocin levels in imprinted gene systems. In contrast to such reductions, Williams syndrome, which is due to hemizygous deletion of about 20–25 genes on chromosome 7 in humans, provides evidence regarding imprinted gene effects that involved increased levels of oxytocin. This syndrome thus involves ‘hypersocial’ behavior, with overdeveloped social motivation and treatment of strangers as familiar friends (Järvinen et al. 2013), with a hormonal correlate in greatly increased levels of plasma oxytocin (Dai et al. 2012). Indeed, a wide range of the behavioral, cognitive, and neurological phenotypes of Williams syndrome closely parallel the effects of oxytocin administration to healthy individuals, for such traits as increased social approach (Kemp and Guastella 2011; Järvinen et al. 2013), trust (Zhong et al. 2012; Godbee and Porter 2013), increased gaze toward faces and eyes (Guastella et al. 2008; Porter et al. 2010), and increased empathy (Fidler et al. 2007; Hurlemann et al. 2010).

In Williams syndrome, behavioral effects have been linked with deletion of the GTF2I gene (Sakurai et al. 2011), and GTF2I, although it has not been demonstrated to be imprinted, demonstrates a strong maternal-chromosome bias in expression (Collette et al. 2009), such that reduced gene dosage (as in Williams syndrome, compared to control individuals) creates a paternal bias in expression and behavior. This syndrome thus appears to represent, in part, the converse of situations where oxytocin is decreased due to loss of paternal expression (as for PEG3 and NDN), and it implicates oxytocinergic effects of imprinted genes even more broadly in human social behavior and cognition.

Despite the considerations discussed above, the degree to which genomic imprinting conflicts impacting the oxytocin system mediate human health and disease remains incompletely understood. Increasing evidence, however, indicates that early development of the oxytocin system in humans exerts lifelong effects on social behavior, stress, subjective well-being, and risk for a broad swath of psychiatric conditions (Cochran et al. 2013; Bethlehem et al. 2014). Analyses of this system that take account of genomic conflicts should yield more comprehensive and more clearly explicable results, as well as elucidating mechanisms that provide new opportunities for health promotion and development of novel therapies. Indeed, in MAGEL2-knockout mice, a single postnatal injection of oxytocin is sufficient to rescue infant feeding deficits (Schaller et al. 2010), and in humans, failure to establish effective breastfeeding is the main cause of clinic visits by mothers and infants (Wells 2003).

## Sexual antagonism

Conflict of interests that involve genomic imprinting are sometimes referred to as parental antagonism, in that the bulk of such conflicts center on the divergent interests of paternally inherited and maternally inherited genes, within offspring or other individuals subject to asymmetric relatedness of paternal and maternal genes to social interactants (Haig 2013; Haig et al. 2014). In contrast to parental antagonism, evolutionary conflict of interests between the sexes are commonly referred to as sexual antagonism, or intralocus sexual conflict (van Doorn 2009; Pennell and Morrow 2013) (Fig.1B,C).

**Figure 2:**
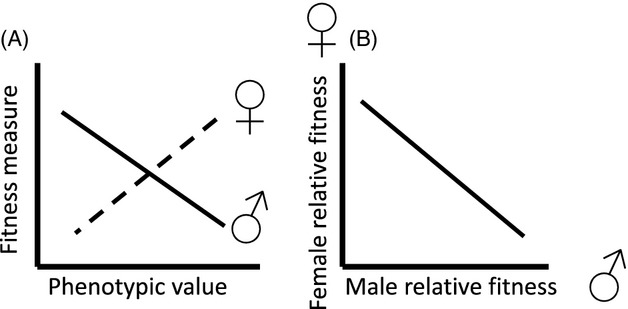
(A) Phenotypic selection gradient comparing trait values of female (dotted line) against male (solid line) fitness. In this example, selecting for higher trait values increases female fitness, but decreases male fitness. (B) Here, sexually antagonistic selection translates to a negative genetic correlation (*r*_mf_) between the fitness of females and males, such that genes that confer high fitness to females will confer low fitness to males, and vice versa.

Sexual antagonism can be defined as a situation whereby females and males express a trait with the same or highly overlapping genetic basis but with different fitness optima (Bonduriansky and Chenoweth 2009) that are not jointly achieved by both sexes. In such circumstances, alleles are sexually antagonistic to the extent that they increase the fitness of one sex but decrease the fitness of the other (Fig.2). Sexual antagonism has been predicted from simple models to represent a pervasive impediment to phenotypic optimization by selection (e.g., Connallon and Clark 2014a), yet its expected and apparent impacts on human health and disease have seldom been investigated (Morrow and Connallon 2013).

Sexual antagonism differs from parental antagonism (genomic imprinting) in several important ways. First, parental antagonism involves genomic conflict between two parties (types of genes) within the same individual, which can be considered as a single genomic ‘niche’ for interacting genes (Patten et al. 2013). As a result, optimal trait values cannot be jointly achieved for maternal genes and paternal genes at the same time, so the conflicts cannot be resolved in any evolutionary sense unless one side irrevocably wins. By contrast, sexual antagonism involves two different ‘niches’ for genes, males and females, such that the evolution of fully sex-specific trait expression, where each sex reaches its optimum, is feasible in principle and could resolve the conflict, at least in the short term (Pennell and Morrow 2013; Haig et al. 2014).

Second, whereas parental antagonism generates organism-level maladaptations along an axis of resource demands imposed by offspring on mothers during development (affecting physical and psychological resource acquisition), sexual antagonism is predicted to generate sex-specific forms of maladaptation along an axis of male–female differences, such that male trait values will be displaced toward (or away from) those of females, and vice versa, each to some degree. As for genomic imprinting effects, deviations from optimal phenotypes are expected to manifest primarily as deviations in health, such that particular alleles increase the health of one sex but decrease health of the other, or decrease the health of both. Third, sexually antagonistic alleles and effects are expected to be concentrated on sex chromosomes (the X and Y in mammals), because of the different inheritance patterns of the sex chromosomes and autosomes. Recent theoretical work has demonstrated that X-linked genes should favor females, while autosomal genes should favor male fitness optima (Frank and Crespi 2011; Connallon and Clark 2011; but see also Patten and Haig 2009 for a model predicting male-biased alleles on the X chromosome). Furthermore, the transmission of sex chromosomes also leads to different levels of relatedness along lineages when comparing males and females relative to their ancestral sex chromosomes (Rice et al. 2010). Empirical work from *Drosophila* indicates that the X chromosome is a hotspot for sexually antagonistic genes (Gibson et al. 2002; Innocenti and Morrow 2010), although it is currently unclear whether this finding applies to species, such as mammals, that possess a higher ratio of autosomal to sex chromosomal DNA and also differ in the nature of dosage compensation. Most generally, to the extent that sex chromosomes represent foci for sexually antagonistic effects, they may also differentially mediate the health effects of such conflicts, unless antagonisms are also more readily resolved to sex-specific optima for sex chromosomal genes than for autosomal genes.

In this review, we focus on the classic scenario of sexual antagonism, in which opposing directional selection acts on a trait to create an evolutionary tug-of-war for shared alleles between the sexes. We note, however, that there are several potential relationships between genotype, phenotype, and fitness that can reflect the operation of sexual antagonism; for example, alleles with sexually dimorphic or opposing phenotypic effects can result in one sex achieving and the other sex overshooting its fitness optimum (Turelli and Barton 2004; Connallon and Clark 2014b) even in the absence of sex-differential selection. Information on the specific contexts and forms of selection on phenotypes, and genetic basis of phenotypic variation in each sex, are thus ultimately required for a thorough understanding of how sexual antagonism impacts human health and disease.

## Detection and measurement of sexual antagonism

How common and important is sexual antagonism in nature, and how do its consequences affect risk and forms of human disease? Demonstration of sexually antagonistic selection for a given trait requires that several criteria be met.

First, the two sexes must differ in fitness optima for some phenotype, with a shared genetic basis between males and females. With regard to hormonally mediated traits, Table2 provides a suite of evidence, for humans and other mammals, that the two sexes not only differ but show opposite phenotypes in response to some hormonally mediated stimulus. To the extent that either males or females deviate from their sex-specific fitness-maximal responses in such circumstances, such opposite effects may represent especially likely conditions for sexual antagonism, because, unless male and female responses are uncoupled, a favorable allele that augments a response in one direction, for one sex, should necessarily be unfavorable in the other sex.

**Table 2:** Evidence of directly-opposite associations between nonapeptide hormones and outcome variables, in males compared to females

Species	Findings	Citation(s)
Human	Intranasal AVP administration is associated with decreased perception of unfamiliar same-sex faces as friendly in males; increased perception as friendly in females	Thompson et al. (2006)
Humans	Intranasal oxytocin administration decreases amygdala responses to fearful faces in males, increases them in females	Domes et al. (2007a,b) and Domes (2010)
Humans	Intranasal oxytocin administration increases left amygdala responses to mutual cooperation in males, decreases them in females	Rilling et al. (2014)
Humans	Intranasal oxytocin administration decreases sympathetic nervous system responses and emotional arousal in females, increases them in males	Ditzen et al. (2013)
Humans	Males rate neutral faces more negatively after intranasal oxytocin administration; females rate them more positively. Males make more negative social-judgement ratings after oxytocin; females make more positive ones	Hoge et al. (2014)
Humans	Intranasal AVP administration increases friendliness perception in females, decreases it in males	Thompson et al. (2006) and Uzefovsky et al. (2012)
Humans	Intranasal oxytocin administration followed by social stress induction decreases anger in males, increases it in females; over time, oxytocin decreases positive mood in females, increases it in males	Kubzansky et al. (2012)
Hamsters	AVP injections into hypothalamus increase aggression in males, decrease it in females	Albers (2012)
Rats	Injection of AVPR1a receptor antagonist into brain reduces social play in males, increases it in females	Veenema et al. (2013)
Finches	Knockdown of AVP production increases aggression towards opposite sex in males, decreases it in females	Kelly and Goodson (2014)

Second, as noted above, selection must be opposite in direction between the sexes for a sexually antagonistic trait, such that an adaptive allelic effect in one sex is associated with a maladaptive effect in the other sex. Assessment of such effects is commonly carried out through measurements of phenotypic selection (such as selection gradient analyses) that examine a phenotypic value against a measure of fitness, partitioned by sex (Fig.2). Various fitness components have been used to quantify this pattern, with lifetime reproductive success providing the most evolutionarily salient information. Counting numbers of offspring provides a simple and straightforward proxy of lifetime reproductive success that also allows for cross-species comparisons (Jones 2009) and yields an indication, given genetic information on the bases for the phenotype, of how genotypic frequencies might be expected to shift in a population across generations.

Several specific approaches have commonly been used to detect and quantify effects of sexual antagonism. First, within sibships, segregating sexually antagonistic alleles should tend to increase the fitness of one sex but decrease the fitness of the other sex. Such effects are expected to manifest both in terms of components of fitness, such as numbers of offspring, and in terms of risks and severity of sex-associated diseases that are observed or predicted to decrease fitness. Such family-based data can also be used to compare father–daughter and mother–son fitness measures using regression methods (Falconer and MacKay 1996; Foerster et al. 2007), with sexual antagonism indicated by lower fitness of cross-sex, compared to same-sex, parent–offspring pairs. These approaches are notably amenable for use with large epidemiological databases that include pedigrees as well as health information, as described in more detail below for the case of schizophrenia.

Second, quantitative genetic methods have been used to infer the operation of sexual conflict, given that this process is expected to generate negative genetic correlations between the fitness of males compared to females (i.e., genetic trade-offs; Robinson et al. 2006; Foerster et al. 2007; Schroderus et al. 2010). This approach, which is amenable for use with multiple-generation pedigree data, represents a generalization of the simple family-based methods described above and can be used to explore the impacts of sexually antagonistic gene expression over more than one generation. Such genetically based life-history perspectives can be powerful tools for quantifying genetic conflict and other traits affecting health and disease in humans (Stearns and Koella 2008; Gluckman et al. 2009), because they are designed to elucidate the sources, mechanisms, and effects of genetically based trade-offs.

Third, artificial selection experiments that involve directional selection on one sex for specific sex-associated traits, and tracking of phenotypes and measures of fitness in offspring of both sexes, have been used to test predictions regarding sexually antagonistic effects (e.g., Mills et al. 2012; using bank voles). Such selection experiments may also focus directly on the sex-differential fitness consequences of putative sexually antagonistic alleles, by breeding or selecting individuals for specific candidate genotypes and determining the sex-specific effects on associated phenotypes and components of fitness, preferably in relatively natural environments (Mokkonen et al. 2011). These methods generate experimental models of how sexual antagonism may be operating in the field, whose results can be compared with patterns in natural populations. Research on *Drosophila* genetics has provided the most extensive experimental evidence for sexually antagonistic genes through such selective breeding, in the laboratory environment (e.g., Rice 1992; Chippindale et al. 2001), as well as via studies of drift in small populations (Hesketh et al. 2013). A simple extension of this general approach, which has yet to be employed, involves testing for sex-specific positive selection in natural populations and determining the phenotypic and fitness effects of the derived and ancestral alleles in males and females. This method would be especially useful, and straightforward, for human populations and for connecting sexually antagonistic alleles with consequences for health.

Fourth, sex-biased patterns of gene expression have been used to test for effects of sexual antagonism and associations of sexual antagonism with the relative strengths of selection on males compared to females (Connallon and Knowles 2005; Innocenti and Morrow 2010; Griffin et al. 2013; Pointer et al. 2013). For example, evolution under enforced monogamy in *Drosophila* ‘feminizes’ gene expression patterns of both sexes, which is expected to favor female optima (Hollis et al. 2014); might similar processes have typified human evolution and account in part for the male-biased incidence of most diseases?

Finally, theoretical predictions regarding expected concentrations and effects of sexually antagonistic alleles on sex chromosomes can be leveraged to test for sexual conflict (e.g., Mank and Ellegren 2009; Allen et al. 2013), given, for example, that alleles of benefit to females should be found differentially on the X chromosome in mammals and that sexually antagonistic drive should generate genomic conflicts, and harmful fitness-reduced effects, among opposite-sex siblings (Rice et al. 2008, 2010).

To evaluate the impacts of sexual conflict on human health, the approaches described above must be dovetailed with information on sex differences in genotype–phenotype associations that are relevant to risks and manifestations of disease (Ober et al. 2008; Gilks et al. 2014). Such dimorphisms are expected to be driven by sexual differences in gene expression (and other allelic effects) that trace, ultimately, to the causes of human sexual differentiation. Steroid and peptide hormones represent among the most important such causes, and as such, they should be the primary mediators of sexual conflict in its influences on human health and disease. How, then, might sexual antagonism be mediated by genetically based effects of hormonal variation?

## Sexual antagonism and genetically based hormone systems

In changing social and abiotic environments, hormones provide effective mechanisms for regulating behavioral responses appropriate to a given sex, situation, and stimulus. Selection should thus favor different regulatory phenotypes specific to each sex (Clutton-Brock 2007; Kokko and Jennions 2008), yet responses to selection may be constrained due to the largely shared genome.

As described above, hormones provide primary mechanisms for development, physiology, and behaviors to operate in context-specific and sex-specific manners. For example, plasma testosterone is found in much higher concentrations in males, while females demonstrate higher concentrations of oxytocin, at least in most studies (Altemus et al. 1999; Carter 2007; Pierrehumbert et al. 2010; Weisman et al. 2013). Currently, testosterone is the best studied hormone with regard to evidence for sexual antagonism in humans and other animals, and the developmental and physiological mechanisms that underlie such effects are relatively well understood (Ketterson et al. 2005; Stearns and Koella 2008; Hau and Wingfield 2011). The role of testosterone in mediating sexual antagonism is further bolstered by the fact that the androgen receptor gene, which is important for the functional response to testosterone, is located on the X chromosome in humans.

We next describe evidence for sexual antagonism, especially as mediated by effects of testosterone (and related steroid hormones), in non-human animals, and we briefly relate this evidence to human health and disease. We then focus directly on evidence regarding sexual antagonism in humans, in the context of the methods discussed above, and discuss the relevance of such evidence to disease. We concentrate specifically on several areas of central importance to human health: life-history phenotypes, behavior, immune function, and psychology.

## Sexual antagonism, testosterone, and life-history traits

The organizational and activational effects of testosterone on physiological and behavioral traits make it an important mediator of vertebrate life-history traits and provide a core context for effects of sexual antagonism. The influence of this hormone can thus mediate the timing of reproduction, the growth and development of offspring, and, most importantly, sex-specific traits that affect components of survival and reproduction.

A series of studies of bank voles (*Myodes glareolus*) has demonstrated that the physiological and behavioral effects of testosterone confer advantages in male–male competition: Males with higher circulating plasma testosterone levels achieve greater reproductive success (Mills et al. 2007, 2009). However, there is also direct sexual antagonism over testosterone levels, whereby responses to artificial selection increase the fitness of males at the expense of females through antagonistic effects on mating behavior (Mills et al. 2012; Mokkonen et al. 2012). As testosterone is known to affect sexual motivation and dominance behavior in mammals, males will, to a point, achieve greater mating success with greater testosterone production. The sexual antagonism is realized because female propensity to mate has been shown to decrease with selection for testosterone; brothers and sisters thus cannot both benefit from selection for testosterone. At the population level, the benefit that testosterone confers on males in intrasexual competition is highly dependent on the social environment of individuals, as too many dominant males in the population will result in lower average reproductive success (Mokkonen et al. 2011); this frequency dependence can maintain genetic polymorphisms when there are competing sex-specific optima in a population. Such patterns may also be influenced by age: A quantitative genetic study on bank voles (M. Mokkonen, E. Koskela, T. Mappes and E. Schroderus, unpublished data) has shown that sexual antagonism over fertility increases with the age of females, presumably due to the sex-differential declines in fertility that are mediated by sex hormones. Potential parallels with human life history are of interest because the reproductive life span of females appears to be increasing over time (Stearns et al. 2010); older females are predicted to express sexually antagonistic alleles more strongly, which may influence risks of reproductive disorders in particular.

Observations of other mammalian species have provided notable evidence of sexual antagonism in natural environments, with parallels to the well-studied bank voles described above. Thus, for example, red deer males that had higher fitness in terms of lifetime reproductive success sired daughters that generally had lower fitness (Foerster et al. 2007). Likewise, Soay sheep experience sexually antagonistic selection over horn phenotype (Robinson et al. 2006). Body mass in mountain goats has also been found to be consistent with sexual antagonism, given that paternal body mass is negatively correlated with daughter's body mass, but positively correlated with son's body mass (Mainguy et al. 2009). Finally, data on bighorn sheep show that longer-horned males sire sons with higher viability, but daughters with lower viability (Martin et al. 2014); such effects appear to be mediated in part by effects of male testosterone levels on social rank (Martin et al. 2013).

What are the expected consequences of such findings for human health and well-being, given that such sexually antagonistic effects on physiological, behavioral, and life-history traits are commonly found across mammals? The most general predicted phenotypic pattern would be that male-beneficial effects of higher testosterone would be associated with lower health and fitness among females; conversely, female-beneficial effects of hormonal phenotypes that differentially involve benefits on females (such as those involving higher estradiol, and oxytocin) would be expected to have negative impacts upon males. As described in more details below, patterns consistent with this expectation have been reported for some human phenotypes, although only a few of them have been directly analyzed for association with physical or psychological health. Additionally, there is considerable opportunity for including models and predictions of sexual antagonism to understanding pathology of fertility-related diseases. Given that sexual antagonism results in reduced reproductive success in one sex, these fitness costs may differentially manifest in disease-related aspects of reproductive physiology, among both males and females.

## Sexual antagonism, testosterone, and immune function

One of the key features of testosterone that has drawn the interest of evolutionary biologists is the pleiotropic nature of this hormone in mediating several important fitness-related functions and trade-offs (Hau and Wingfield 2011). Immunosuppressive effects of testosterone production and response sensitivity have been a central focus of research in this area (Folstad and Karter 1992; Mills et al. 2010; Hau and Wingfield 2011), in that they apparently represent a fundamental life-history trade-off that may render some individuals more susceptible to disease. To the extent that males benefit more than females from testosterone production, and if such production is subject to effects of sexually antagonistic alleles, then females may be hypothesized to differentially suffer immune system costs from relatively high testosterone.

Evidence that testosterone and immune function are subject to effects of sexual antagonism comes from a series of studies of bank voles. Thus, Mills et al. (2010) showed that male voles subject to artificial selection on immune function showed correlated responses to selection in levels of testosterone. Experimental administration of testosterone to males also led to reduced immune function (in the highest testosterone group), as well as increased social status and reproductive success (Mills et al. 2010). Most importantly, Schroderus et al. (2010) demonstrated, using quantitative genetic methods, that immune function and testosterone levels were subject to negative genetic correlations in both males and females, such that selection for higher testosterone in males will result in reduced immune function among females.

Among humans, activity and vigor of the immune system is notably higher among females than males (Bouman et al. 2005; Oertelt-Prigione 2012), and negative associations have been demonstrated between testosterone levels and immune system activity across many studies (Muehlenbein and Bribiescas 2005). The medical relevance of such associations is demonstrated, for example, by lower antibody responses to influenza vaccination among males with higher serum testosterone, in conjunction with overall lower responses among males compared to females (Furman et al. 2014). The degree to which such patterns are genetically based in humans remains unknown, but if genetic architecture in humans resembles that in voles, then sexually antagonistic alleles may be segregating in humans that lead to increased testosterone in males but decreased immune function among females, or both sexes, and vice versa, at least among individuals in relatively poor physiological condition. The presence of such allelic effects would have important medical implications, especially with regard to understanding sex differences in disease risks (Gilks et al. 2014) and their mechanistic, as well as evolutionary, causes.

## Sexual antagonism in human populations

Studies focusing on human sexual antagonism are limited but increasing. Stulp et al. (2012) recently used long-term cohort data to demonstrate intralocus sexual conflict on human height, showing that sisters had higher reproductive success (numbers of children) than their brothers among shorter sib-pairs, but brothers had higher success than sisters among average height pairs. Given evidence of a negative association between height and reproductive success among females, but a stabilizing-selection pattern among males favoring average height, these data indicate that neither sex is expected to achieve its sex-specific optimum for this trait.

A study by Bolund et al. (2013) using pedigree data from a pre-industrial Finnish population highlighted the usefulness of combining phenotypic selection gradient analysis with genetic data to assess sexual conflict in human populations. In this population, the phenotypic selection gradients were opposite in direction between the sexes for first and last reproduction, and reproductive life span, but these traits also showed positive, rather than negative, genetic correlations between the sexes, with regard to fitness (Bolund et al. 2013). Sexual antagonism was therefore not realized in this case, despite selection operating antagonistically between the sexes. By contrast, Stearns et al. (2012), using a combination of phenotypic selection data and information on genetic correlations, determined that sexually antagonistic selection was acting on several important human health measures such as height, weight, blood pressure, glucose levels, cholesterol levels, and age at first birth, and constrained joint evolutionary responses to selection in a manner expected to exert impacts on sex-specific human health and disease.

Additional evidence has emerged, for various nonmedical traits, that hormones are implicated in human sexual antagonism. Garver-Apgar et al. (2011) demonstrated that masculine males and females reported higher ‘mate values’ for brothers relative to sisters, suggesting that the influence of higher testosterone increased perceived mate value in males, but resulted in lower perceived mate value for ‘androgenized’ females. Studies of facial attractiveness scores between siblings added support to the hypothesis that sexual antagonism operates in such situations, apparently mediated by effects of testosterone (Manning et al. 2000; Mitchem et al. 2014). These studies would represent effects of sexual antagonism to the extent that perceived mate values were associated with differential fitness by sex in sibships, and especially if such facial-feature and mate-choice effects were linked with hormone-associated genetic variation.

Hormonal effects on human phenotypes have also commonly been indexed by finger 2D:4D ratio, whereby a lower ratio reflects greater prenatal testosterone exposure relative to estradiol, and thus, greater ‘masculinization’ (Lutchmaya et al. 2004). Manning et al. (2000) demonstrated that measures of male reproductive success were higher for individuals with lower (more ‘masculine’) 2D:4D ratios, while the opposite was true for females. This finding was replicated in a larger study (Manning and Fink 2008), but not in a sample of women from Finland (Helle 2010). Although further data are needed, especially regarding the mechanisms linking 2D:4D with measures of fitness, the former two studies are consistent with sexual antagonism effects mediated by prenatal ratios of testosterone to estradiol. Such evidence for antagonism effects is important for health given the evidence for associations of 2D:4D ratios with risks for diseases such as cancer (e. g., Muller 2013) and coronary artery disease (e.g., Wu et al. 2013). In principle, sexual antagonism theory thus predicts opposite effects on males and females in such disease risks versus protection, mediated by loci that influence production and sensitivity to steroid hormones especially during early development.

Finally, human handedness shows one of the primary signatures of sexual antagonism: Female mixed-handers report themselves as more masculine, and male mixed-handers report themselves as more feminine, compared to more lateralized individuals (Tran et al. 2014). Such effects appear to be mediated in part by genetic variation in the androgen receptor, in that among females, left handedness is associated with a larger number of microsatellite repeats, but in males, it is associated with fewer repeats (Medland et al. 2005; see also Hampson and Sankar 2012).

## Sexual antagonism and human behavior, psychology, and psychiatric conditions

A primary signature of sexual antagonism in human behavior-related phenotypes, from evidence available to date, is increased fitness of one sex, combined with decreased fitness of related individuals of the other sex, in the context of a genetically based, sexually dimorphic trait whose expression is mediated by hormones. As described in detail below, this pattern has been reported and substantially replicated in the context of two human phenotypes: sexual orientation and schizophrenia. The former phenotype is not, of course, directly associated with health, although it may be indicative of the potential for sexually antagonistic effects in behavioral and psychological traits linked with risk of disease. By contrast, schizophrenia and the related psychiatric conditions, such as bipolar disorder, major depression, and borderline personality disorder, represent among the most important human health conditions with regard to personal, social, and economic impacts. New perspectives on the causes of these conditions from the evolutionary theory of sexual antagonism should offer novel insights into understanding them and driving strategies for the collection of new data.

A series of ten studies, across multiple ethnicities, has provided evidence supporting a hypothesis of increased fecundity in maternal (but not paternal) relatives of homosexual men (review in Ciani and Pellizzari 2012). Concomitant reduced offspring production of homosexual men, and evidence that some degree of X chromosome linkage for underlying alleles is most compatible with the available genetic association and pedigree data, has led Ciani and colleagues (Ciani et al. 2008) to infer that a hypothesis of sexually antagonistic alleles provides the best explanation for this otherwise-paradoxical trait (Iemmola and Ciani 2009; Ciani and Pellizzari 2012; Ciani et al. 2012). The genetic and environmental mechanisms that mediate such effects remain unclear and complex, although hormones (Balthazart 2011) or X-linked epigenetic factors (Rice et al. 2012) appear to play some roles.

A notable health implication of this pattern of results follows from the finding that female relatives (mothers and maternal aunts) of male homosexuals exhibited significantly reduced complications in pregnancy and fewer gynecological disorders, than did female relatives of heterosexual men (Ciani et al. 2012). Determining the mechanisms that mediate such positive effects should have direct implications for female reproductive health and for health-related pleiotropic effects of the relevant causal factors in homosexual men. More generally, these findings represent clear evidence for sexual antagonism in human development, cognition, behavior, and reproduction and indicate that its effects are likely to also promote the maintenance of variation in more directly health-related traits.

A series of studies on the epidemiology of schizophrenia has focused on the hypothesis that fitness-related benefits in the relatives of schizophrenics may help to explain the high prevalence of this disorder, and its substantial heritability, in the context of its strongly negative impacts on reproduction by patients (van Dongen and Boomsma 2013). None of these studies have reported fitness benefits to relatives sufficiently strong (under realistic models) to maintain schizophrenia risk alleles, but a notable number of them (Bassett et al. 1996; McGrath et al. 1999; Haukka et al. 2003; Weiser et al. 2009; meta-analysis in Bundy et al. 2011) have found evidence that female relatives of individuals with schizophrenia exhibit significantly higher fertility than female control individuals, even though male relatives of schizophrenics show greatly reduced fertility. Most recently, a large study using a Swedish database of over two million individuals (Power et al. 2013) demonstrated strong effects supporting these smaller, more heterogeneous studies: Sisters of schizophrenic individuals had significantly increased numbers of children compared to controls (whereas brothers had significantly fewer); moreover, sisters of individuals with bipolar disorder also showed significantly increased fecundity (brothers showing no difference), and both sisters and brothers of individuals with major depression demonstrated such increases. This study and virtually all previous reports have also shown that schizophrenia itself has substantially more adverse effects on male than female reproduction; this disorder is also more deleterious in its effects on cognitive abilities among males than females (e.g., Abu-Akel and Bo 2013). Such sex differences in schizophrenia prevalence and severity have usually been attributed to protective effects of estrogen in females, although influences from other hormones appear also to be involved (reviews in Mendrek and Stip 2011; Hayes et al. 2012). Additional evidence consistent with effects of sexual antagonism in schizophrenia risk comes from functional-imaging studies that demonstrate ‘masculinization’ of female schizophrenia patients and ‘feminization’ of male patients (Mendrek 2007; Mendrek et al. 2007), mediated in part by levels of testosterone (Mendrek et al. 2011), which suggests dysregulation directly along a male–female axis.

Considered together, these data indicate that schizophrenia demonstrates clear evidence of risk mediation, in part, by effects of sexual antagonism. As such, some notable proportion of schizophrenia risk alleles should exhibit beneficial effects among females (with regard, presumably, to functions ultimately linked with reproduction, and associated cognitive–emotional processes) in addition to their deleterious effects among males (with regard to schizophrenia risk, and its associated, underlying phenotypes). A primary implication of these results is that studies of schizophrenia that do not account for sex differences in the genetic and gene by environment bases of risk will yield results that are at best incomplete, and at worst incorrect. The findings also suggest that the X chromosome, as a nexus for sexually antagonistic effects, may exhibit an especially important influence on schizophrenia risk, as a suite of previous studies has also suggested (e.g., Crespi 2008; Crow 2008; Goldstein et al. 2011, 2013).

The degree to which the patterns of findings described above, for sexual orientation and schizophrenia, generalize to other health-related conditions remains unknown, but relevant theory predicts that any human disease showing sex differences in incidence, severity, or manifestations should be a candidate for genetic risks mediated in part by sexual antagonism. Among psychiatric conditions, autism demonstrates among the strongest evidence for such sex differences and potential effects of sexual antagonism, with a striking male bias in prevalence, clear hormonal effects on risk, greater severity among females, effects from X-linked loci, and disorder-associated enhancements in some performance traits related to perception, visual-spatial skills, and mechanistic cognition, which are concentrated among males (Marco and Skuse 2006; Mottron et al. 2006; Treffert 2009; Baron-Cohen et al. 2011; Robinson et al. 2013; Teatero and Netley 2013). As such, genetic risk of autism may engender hormone-associated alleles that tend, overall, to benefit males, but also tend to increase risk of autism and other testosterone-linked diseases among females. This hypothesis is supported by evidence for elevated incidence of testosterone-related disorders in the female relatives of autistic individuals (Ingudomnukul et al. 2007) by high rates of testosterone-related ‘steroidopathies’ among women with autism spectrum disorders (Pohl et al. 2014) and by higher levels of serum testosterone in females (but not males) with autism, compared to controls (Schwarz et al. 2011; Bejerot et al. 2012). A general prediction of the hypothesis is that some ‘autism risk’ alleles should also confer benefits to males in healthy populations (e.g., in visual-spatial abilities), but also be associated with costs to females.

## Conclusions

One of the primary uses of evolutionary biology in the study of human health and disease is indicating what novel data would be especially useful to collect. Genomic conflicts provide a paradigmatic example of such evolutionary insights, because their dynamics and predictions are well beyond the purview of standard medical frameworks for studying and understanding disease. We have analyzed two of the primary forms of genomic conflicts, genomic imprinting (parental antagonism) and sexual antagonism, because they both predict conflict-driven, health-related deviations from optimal phenotypes for one or both of the parties involved, in the core fitness-related domains of parent–offspring and male–female interactions, respectively. With regard to genomic imprinting effects, we have described evidence, from theory and empirical work, that imprinted genes impact upon development of the human oxytocinergic system, a system that mediates lifelong psychological health and well-being. Thus far, genetic studies of this system have focused almost exclusively on the nonimprinted genes OXTR, OXT, and CD38; our results indicate that genetic, epigenetic, and functional studies of the imprinted genes PEG3, NDN, MAGEL2, DLK1, and GTF2I should lead to important new insights into the roles of oxytocin in human health, especially with regard to infant resource solicitation, mother–infant interactions, and their downstream psychological effects. With regard to sexual antagonism, our review suggests that some notable proportion of human sex-differential risks of disease incidence and severity may be attributable to such conflicts, especially in the context of positive versus negative effects of phenotypes mediated by testosterone, estrogen, and other hormones in males compared to females. As regards analyzing the genetic bases of human disease risks, such considerations should compel data collection and analysis that explicitly tests hypotheses of protection from disease in one sex, but increased risks of disease in the other, attributable to effects from the same locus. In addition to helping uncover new sources of disease risk, the study of sexual antagonism should thus also demonstrate beneficial sex-specific phenotypes, such as increased reproductive health among females or males, due to favorable sex-specific alleles. Studies of human sexual antagonism should dovetail directly with ongoing genetic association, large-scale epidemiological, and selection-measurement analyses, such that testing for sex-antagonistic effects mainly requires increased awareness of its expected prevalence, dynamics, and impacts on risks and phenotypes of sex-differential disease.
